# Maximizing Resource Efficiency in Wireless Networks through Virtualization and Opportunistic Channel Allocation

**DOI:** 10.3390/s23083949

**Published:** 2023-04-13

**Authors:** Esteban Inga, Juan Inga, Roberto Hincapié

**Affiliations:** 1 Department of Master’s Degree in ICT for Education, Smart Grid Research Group (GIREI), Universidad Politécnica Salesiana, Quito EC170525, Ecuador; 2 Telecommunications Research Group (GITEL), Universidad Politécnica Salesiana, Cuenca EC010102, Ecuador; jinga@ups.edu.ec; 3 Information and Communication Technology Applications Research and Development Group (GIDATIC), Universidad Pontificia Bolivariana, Medellín CO050031, Colombia; roberto.hincapie@upb.edu.co

**Keywords:** smart city, cognitive mobile virtual network operator, matching, assignment, optimization, wireless sensor networks, Hungarian algorithm, resource allocation

## Abstract

Wireless cellular networks have become increasingly important in providing data access to cellular users via a grid of cells. Many applications are considered to read data from smart meters for potable water, gas, or electricity. This paper proposes a novel algorithm to assign paired channels for intelligent metering through wireless connectivity, which is particularly relevant due to the commercial advantages that a virtual operator currently provides. The algorithm considers the behavior of secondary spectrum channels assigned to smart metering in a cellular network. It explores spectrum reuse in a virtual mobile operator to optimize dynamic channel assignment. The proposed algorithm exploits the white holes in the cognitive radio spectrum and considers the coexistence of different uplink channels, resulting in improved efficiency and reliability for smart metering. The work also defines the average user transmission throughput and total smart meter cell throughput as metrics to measure performance, providing insights into the effects of the chosen values on the overall performance of the proposed algorithm.

## 1. Introduction

Currently, smart cities aim to enhance the services offered to their citizens. They seek to improve efficiency and reliability while reducing operational costs [1]. However, service providers encounter challenges such as meter readings, cuts, and reconnections of essential services.

Therefore, to address these challenges, this study aims to determine a solution that can impact the costs of communications infrastructure used for smart metering for each basic service. However, creating a heterogeneous network that can be deployed rapidly poses a significant challenge. Using the existing cellular telephony infrastructure with a high penetration percentage in cities provides a viable alternative. This study seeks to demonstrate that a business model based on a cognitive mobile virtual operator (C-MVNO) can reduce the costs of accessing smart meters through cellular technology [2,3].

MVNOs face challenges when utilizing the primary spectrum, such as assigning compatible channels. This study proposes an intelligent metering channel assignment method for MVNOs that considers the reusability of communication infrastructure to reduce maintenance and scalability costs [4,5]. During the simulation, it was observed that the smart meter device installed in households transmits data to the cellular base station via an uplink channel. By using cognitive radio and MVNO, it is possible to detect white holes that may occur in the primary spectrum, enabling spectrum reuse and minimizing the need to lease the secondary spectrum, reducing costs [6,7].

On the other hand, a traditional MVNO accesses the primary spectrum under a long-term contract for the network infrastructure. However, the range must be constant to be profitable, and the lease with the mobile network operator (MNO) can be exploited continuously. Therefore, a C-MVNO may be more useful since it can access or acquire access to the spectrum for short periods by detecting the “spectrum holes” that are free for use, allowing the dynamic lease of the range [8]. However, for the data transport that occurs in smart metering systems, proper channel planning is necessary to ensure the timely delivery of information, reducing problems related to latency and lost packets [9].

The opportunistic use of channels to read a smart meter at specific times helps to maximize channel usage. Similarly, aggregating information from a significant number of smart meters at a stage wherein data compression is possible further improves the performance of the communication infrastructure.

Implementing specialized communication networks for the smart metering of utilities, such as electricity, potable water, or gas, has been a subject of ongoing research. Despite this, few proposals take advantage of the existing cellular network, which boasts a higher penetration rate than a newly established heterogeneous network.

Therefore, to make the use of cellular networks for smart metering financially viable, it is imperative to utilize secondary or opportunistic channels. A cognitive mobile virtual network operator offers the possibility of exploiting areas of low cellular congestion, thereby reducing costs. Alternatively, leasing dedicated channels as a secondary operator for smart metering in regions with high cellular congestion is also feasible. Utilizing an existing network reduces the deployment time, the primary operator is responsible for managing maintenance, and smart meter coverage is guaranteed.

Utilizing an existing cellular network for smart metering presents a promising approach as it offers several benefits over deploying a dedicated communication network, such as extensive coverage, high reliability, and scalability. Previous research has suggested that a cellular network is more cost-effective than a heterogeneous network due to increased deployment and maintenance costs. Therefore, using secondary or opportunistic channels is a viable option that can significantly reduce the cost of smart metering.

In less congested areas, a cognitive mobile virtual network operator can facilitate the deployment of smart metering solutions, which can optimize the available cellular resource and opportunistic features. Dedicated channels can be leased from the primary operator in highly congested areas, reducing the deployment time, ensuring coverage, and lowering maintenance costs.

Scientific papers have highlighted that the smart metering of utilities, such as potable water, electricity, or gas, does not need to be real-time and can be obtained from smart meters at longer time intervals, such as every 7, 15, or even 30 min, as required by the utility monitoring consumption. This recommendation is based on the fact that most utility metering applications do not require real-time metering, and a lower metering frequency does not significantly affect the accuracy of the recorded data [10].

In addition, smart utility metering uses high-resolution sensors and wireless communication technologies that enable the continuous capture and recording of consumption data. It is possible to adjust the metering frequency according to the utility’s specific needs and reduce the amount of data to be processed and stored without compromising the quality of the recorded data. In summary, smart utility metering does not need to be real-time. The metering frequency can be adjusted according to the utility’s needs to reduce the amount of data to be processed and stored without compromising the quality of the recorded data [11].

**Figure 1:**
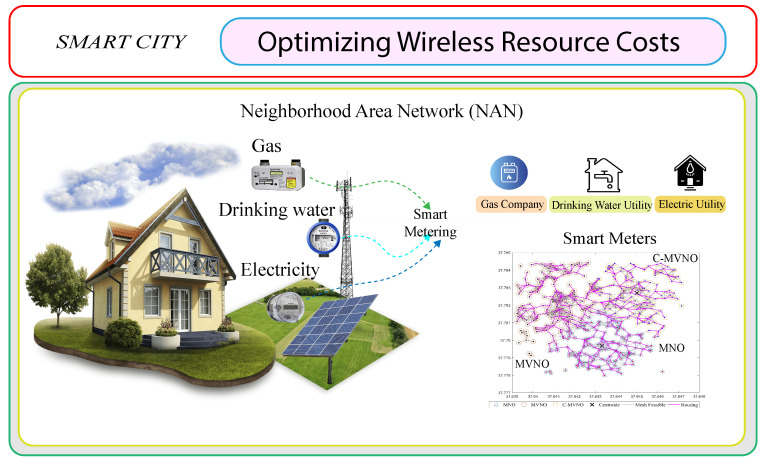
Process diagram of harmonic distortion detection by compressed sensing.

The concept of responding to the smart metering of essential services with the lowest cost through optimizing the available cellular resource and opportunistic features is illustrated in Figure 1. The present work is organized as follows: Section 2 reviews related jobs; Section 3 formulates the problem and discusses the proposed methodology; Section 4 analyzes the results; and Section 5 states the conclusions.

## 2. Related Works

Primary spectrum leasing is typically mentioned when discussing mobile operators, while virtual mobile operators are associated with secondary spectrum renting. Therefore, this paper proposes to use a secondary spectrum for the smart metering of essential services such as drinking water, gas, and electricity, intending to reduce the initial investment and minimize wireless infrastructure deployment. If a mature, consolidated, and scalable infrastructure’s secondary spectrum can be utilized, it is feasible to reduce costs [12,13].

Each central’s meter data management system (MDMS) manages the information, including cuts and reconnections of supplies, smart meter readings, and data processing. When sizing the network for smart metering, it is necessary to strategically consider deploying wireless networks to generate efficient and reliable bidirectional communication between consumers and service providers [14]. This article analyzes only neighborhood area network (NAN) deployment with channel reuse [15,16].

The cognitive virtual mobile operator (C-MVNO) represents an evolution of the MVNO concept that utilizes cognitive radio to optimize channel usage, thereby reducing the cost of leasing the primary spectrum from the operator. Traditional MVNOs that previously had to lease the spectrum from the primary operator can now allocate less of the spectrum through C-MVNO by finding white holes in the primarily licensed spectrum band. The Hungarian algorithm facilitates the optimal matching of resources [17,18]. The dynamic channel allocation of secondary spectrum channels through C-MVNO minimizes leasing costs by enabling the flexible and opportunistic use of the track based on the wireless traffic demands of unlicensed secondary smart metering users. Therefore, it is necessary to ensure that MVNOs obtain enough wireless resources to serve their users and meet their QoS requirements, guaranteeing to maximize the utilization of the discovered resources [19,20].

Assuming that detecting the available spectrum is less expensive than paying for a permanent spectrum lease, the capacity of the spectrum to be used is probabilistic due to the random access of mobile terminals to the primary range [21,22]. Consequently, when assigning secondary spectrum use to another MVNO, the detection of white holes becomes more challenging [23,24].

Traditionally, leasing has been required for a specific range of channels reserved for smart metering and finding white holes to transmit information and minimize leasing costs. This leasing ensures that data are recovered over guaranteed media during network congestion [25,26]. Algorithms such as the Hungarian one can analyze channel allocation for a dedicated C-MVNO for intelligent metering services, maximizing the number of innovative meter readings and optimizing channel use over an opportunistic scenario [27,28].

Under these circumstances, employing a business strategy that reuses the communications infrastructure, such as MVNO, necessitates research to ensure economic viability in the long term. It is also vital to consider network scalability to minimize technological and economic impacts [29,30].

The history of this C-MVNO refers back to the need for smart electric power, potable water, or gas metering in hard-to-reach areas or urban areas where infrastructure installation is costly. The proposed C-MVNO model solves this problem by leveraging existing infrastructure and wireless technology to perform metering efficiently and economically. In addition, an innovative business model is proposed to minimize the costs associated with the infrastructure and make the service more accessible and sustainable [31,32,33].

This scientific paper represents a significant innovation in wireless resource allocation for smart metering services in a C-MVNO. Unlike other proposals, the Hungarian algorithm is employed for the more efficient allocation of wireless channels. A Markov chain simulates cellular connection times, allowing for a more accurate and realistic channel capacity evaluation. In addition, using reserved and secondary user channels for areas with different traffic provides a flexible and efficient strategy for resource management [34].

Therefore, to achieve the efficient manipulation of the random channel spectrum in a frequency-constrained C-MVNO, it is necessary to implement opportunistic allocation and virtualization techniques. Opportunistic allocation allows the use of free and available channels at specific times. In contrast, virtualization enables the creation of multiple virtual instances of the system that share the same physical resources. It allows for better use of the channel spectrum and greater efficiency in wireless resource allocation [1,35,36].

Although opportunistic wireless channel allocation and virtualization are promising techniques for resource management in a C-MVNO, there are challenges and limitations associated with the random access of mobile terminals to the primary range. More advanced and efficient resource management algorithms must be developed to address these challenges and ensure optimal and sustainable resource management.

This C-MVNO specializes in wireless resource management and efficient channel allocation for smart metering services. Unlike other scientific papers that focus on resource allocation in a general way, this paper focuses on the specific allocation of wireless channels for the smart metering of electric power, potable water, or gas. In addition, using the Hungarian algorithm and Markov chain enables the more efficient and realistic allocation of wireless resources, significantly improving the efficiency and quality of service.

In summary, Table 1 displays the recently evaluated fields regarding the topics discussed in this paper. The innovation presented in each proposal has contributed significantly to the need to reduce costs for the intelligent metering of essential services.

## 3. Problem Formulation and Methodology

The task involves deploying a wireless cellular network via a primary network operator, which provides voice and data access to cellular users via a grid of cells. Each cell is equipped with a set of carefully selected channels so as not to interfere with those of neighboring cells. Additionally, several smart meters are randomly placed within the same area as the cells. The channel allocation process is assumed to be Markovian.

In this scenario, each cell contains a set of smart meters that periodically require an uplink channel. The responsibility for providing service to these smart meters falls on a virtual mobile operator (MVO), which can be an independent entity, such as an electric company. For simplicity, its job will focus on a single-cell model that covers *M* smart meters with *N* available channels.

The primary cellular operator divides its channels into three sub-classes. Firstly, a set of $N_{r}$ reserved channels are exclusively assigned to cellular users. Secondly, there is a set of $N_{f}$ fixed channels rented by the MVO for transmitting information related to the smart meters. Lastly, there is a set of $N_{ra}$ random channels are less expensive than the fixed channels and can be used deterministically if not used by cellular users. The total number of channels available is $N=N_{r}+N_{f}+N_{ra}$.

A time slot is an interval in which the smart meter needs to transmit the information corresponding to the smart meter consumption. In a certain time slot, there may be $n_{c}$ channels used by cellular users guaranteeing that $n_{c}\leq N_{r}+N_{ra}$. It may also schedule $n_{a}$ smart meters to be active during this period. From them, only $n_{a}^{*}$ will transmit, with $n_{a}^{*}=min\left\{n_{a},\;N-n_{c},\;N_{f}+N_{ra}\right\}$.

If the smart meter is scheduled for transmission using a reserved channel, it will successfully transmit at all times. However, if the smart meter is scheduled for transmission over a random channel, it may send or not according to a certain probability. The scenario supposes that the time slot and channel assignment are determined by the primary cellular operator, who will decide the transmission spaces according to the availability of the random channels.

The transmission schedule is defined by a scheduling algorithm SchAlg, which assigns each smart meter the time slot and the channel for transmission. The time slots should be grouped into frames of *T* time slots each.

The scheduling is re-computed for each frame and can be represented by a binary variable $x_{h,t,k}$∈0,1; in fact, $x_{h,t,k}=1$ if the *k*th smart meter uses time slot *t* on channel *h* for transmission; therefore, its scheduling must be bounded by some constraints, such as $\sum_{h=1}^{N}\sum_{k=1}^{M}x_{h,t,k}\leq 1;\forall t\in \left\{1,2,\ldots ,T\right\}$. In this way, only one smart meter is scheduled on the same channel and time slot.

$\sum_{h=1}^{N}\sum_{t=1}^{T}x_{h,t,k}\leq 1;\forall K\in \left\{1,2,\ldots ,M\right\}$, i.e., a smart meter is scheduled for transmission on a single time slot and a single channel. It also supposes that each intelligent meter has a preferred time slot for transmission $t_{k}$, equivalent to a specific phase inside the frame.

However, the smart meter may transmit in a different time slot, within a transmission window $\left\{t_{k}-W/2,t_{k}+W/2\right\}$ with a window length *W* to help avoid congestion. It defines the variable $z_{h,t,k}\leq x_{h,t,k},\;z_{h,t,k}\in \left\{0,1\right\}$, which represents whether the intelligent meter has effectively transmitted information for the scheduled channel and time slot combination.

The scenario defines a set of transmission frames *F*∈1,2,… as well as the quantity $z_{f,k}=\sum_{h=1}^{N}$$\sum_{t_{f}=1}^{T}z_{h,t,k}$ as an indicator equal to one when the *k*th smart meter effectively transmits in the frame *f*. Note that $t_{f}$ is the time slot related to frame *f*.

The scenario defines the average user transmission throughput as

$TH_{k}=\lim_{F\to \inf}\frac{1}{F}\sum_{f=1}^{F}z_{f,k}$. It defines the entire intelligent meter cell throughput by $TH=\lim_{F\to \inf}\frac{1}{F}\sum_{f=1}^{F}\sum_{k=1}^{M}z_{f,k}$. Ideally, the values should have a limit of $TH_{k}\to 1:M$; in fact, these quantities are used to measure performance.

It is well known that the chosen values impact the parameters for the overall performance metrics. Our goal is to find such effects. It is necessary to find the relationship between the performance metrics, as defined, and the system parameters, such as *N*, $N_{f}$, $N_{r}$, $N_{ra}$, and *M*.

Our proposed algorithm for the SchAlg is based on a minimum weight matching problem, where the different intelligent meters are the nodes that should be assigned to a particular time slot/channel combination, which it will call a resource block. This assignment can be seen as a bipartite graph with intelligent meters on a subset and resource blocks on another. The weights and boundaries between nodes are defined next. We define a border between the *k*th intelligent meter and a resource block (h,t) if *t* is between the transmission window of the intelligent meter. The weight, represented by $W_{k}^{t,h}$, is defined according to the following criteria:

- A weight $w_{k}^{t}$ that depends on the distance between the time slot *t* and the preferred time slot $t_{k}$. This minimum weight for the $t_{k}$ value increases with distance to $t_{k}$.
- A weight $w_{k}^{h}$ depends on the chosen channel. If *h* lies on the fixed channel set, it has a lower value. Similarly, if *h* lies on the random channel set, it has a greater value.
- A fair weight $w_{k}$ tries to force proper resource allocation to intelligent meters. $w_{k}$ is a token-based value. If a smart meter has yet to be transmitted on several frames, it accumulates the token matters related to each. When the intelligent meter successfully sends, it adds no value. The $w_{k}$ value is the inverse of the number of accumulated tokens. The smart meter with the more significant transmissions will have a greater weight.

The product of the three weights computes the final weight value. The result is lower than the corresponding smart meter with lower throughput. It will also have a lower value on the time slots closer to the preferred transmission time slot and even lower on the reserved channels.

**Figure 2:**
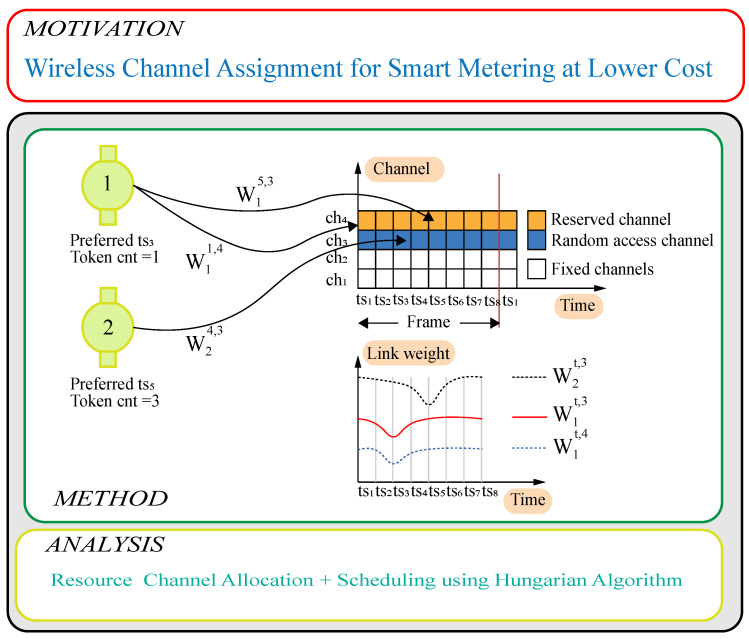
Channel allocation for smart drinking water, gas, and electricity metering.

The weight calculation process is shown in Figure 2. We finally solve the problem as a minimum weight bipartite matching problem by a Hungarian algorithm defining the final scheduling. This scheduling is applied, and the $z_{h,t_{f},k}$ values are computed as input for the next frame. This process is then applied iteratively on subsequent frames. The algorithm is presented in Algorithm 1.

Table 2 describes the variables used in the mathematical model and algorithm.
**Algorithm 1** Scheduling algorithm on frame *f***Require:** M,N,T,W — Input variables**Ensure:** 
TH — Output variable  Step 1Calculate $W_{k}^{t,h}\forall k\in \left\{1,\ldots ,M\right\},\;\forall t\in \left\{1,\ldots ,T\right\},\;\forall h\in \left\{1,\ldots ,N\right\}$;  Step 2Set $x_{h,t_{f},k}$ from solution of minimum weight bipartite matching problem;  Step 3Solve cellular channel occupancy during the frame;  Step 4Set $z_{h,t_{f},k}$ from channel occupancy;  Step 5**forall** k∈{1,…,M}  Step 6    Compute $z_{t_{f},k}$  Step 7    **if** $z_{t_{f},k}==1$ **then** $Token_{k}++$;  Step 8**endforall**

### Methodology and Simulation Process

The methodology for the simulation process is based on the timely allocation of wireless channels for smart metering tasks by sharing the resource with telephony users belonging to the primary and secondary operators. For our analysis, *N* channels the maximum number of users in a cell. Therefore, *N* allows one to calculate the scheduling at a given time if they are busy trying to talk to several users from 0 to *N*. In addition, a Markov chain will represent the value of the distribution of users.

Thus, to create a Markov chain, the algorithm considers two variables, Pa and Pd, as probability values. Pa indicates the probability of having an inactive smart meter initiate a call. On the other hand, Pb is the probability of having an active smart meter end a call.

The simulation considers dividing the available capacity of a mobile operator into $N_{f}$ fixed channels, Na random channels (for MVNOs), and $N_{r}$ reserved channels (for traditional MNOs).

$N_{f}$ is the number of resources available to the primary operator to satisfy its calls, not shared with any MNO or MVNO. $N_{r}$ represents the resources reserved for the virtual operator MVNO. Finally, $N_{ra}$ means that the random channels are not given to the MVNO unless the operator assigns them. These channels make transmission possible when it is not possible with random media.

Therefore, our simulation scenario relies on four parameters to operate: first, the available resources; second, how many channels are reserved for the actual MNO operator; third, the insufficient number of channels dedicated for an MVNO; and fourth, how many channels can be used opportunistically when the primary operator does not use them. These values are $N_{f}$, $N_{r}$, and $N_{r}a$, respectively.

The other parameter analyzed is an intelligent meter read period, denoted T. This period can vary. In several innovative meter projects, T ranges from 15 to 30 min (electricity). The model has a fixed number of time slots. Therefore, if T is very short, it may only read some intelligent meters. However, the system can work if T is fast and the number of reserved channels is significant. It is the set of interrelationships between $N_{r}$, $N_{f}$, $N_{r}a$, and T. Thus, the proposed model aims to indicate the system’s behavior by varying Na and $N_{r}$, leaving the total number of channels and the number of intelligent meters fixed.

Given $N_{r}$, the channels reserved by the MNO are compared against the number of random channels delivered by the MVNO. In other words, the model analyzes the responses of different values of $N_{r}a$ and $N_{r}$. Our scenario sets $N_{f}$ to 10, $N_{r}a$ to 4, and $N_{r}$ to 4.

Figure 2 presents a clear perspective on the intelligent meters competing to transmit over random access channels or the reserved channels (prepay) and the relations with fixed channels (cellular service).

## 4. Analysis of Results

The simulation process has been implemented in a computer with an Intel Xeon 2.6 GHz processor, 128 GB RAM installed, and a 2 TB SSD disk. The software used is Matlab R2022b. The Big Oh notation for the Hungarian algorithm used in the model is $O(n^{3})$.

**Figure 3:**
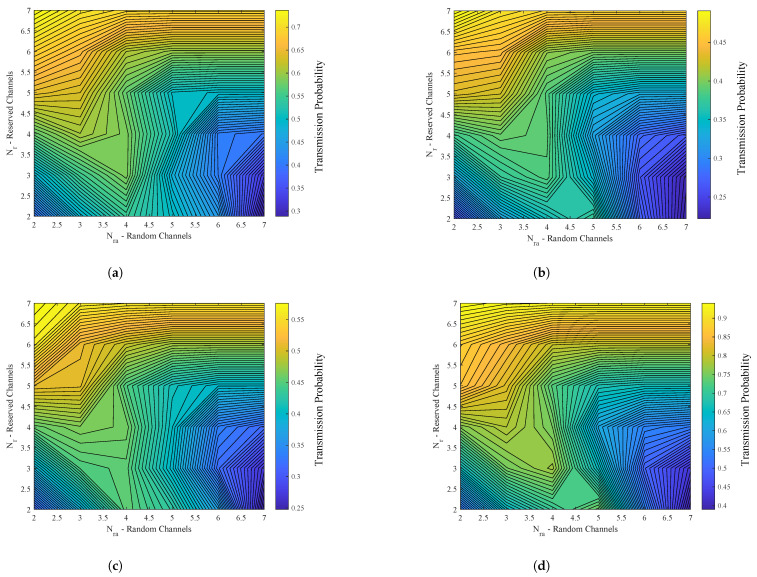
Relationship between $N_{a}$ random channels and $N_{r}$ reserved channels. (**a**) Scenario with 300 smart meters; (**b**) scenario with 250 smart meters; (**c**) scenario with 200 smart meters; (**d**) scenario with 150 smart meters.

Figure 3 shows the relationship between $N_{r}$ and $N_{r}a$, where the color bar determines the transmission probability of the smart meters in an instant of time. The exemplified simulation has N = 10 (channels), M = 300, 250, 200, 150 (smart meters), and T = 15, 20, 30, 45, 60 SM reading time intervals.

**Figure 4:**
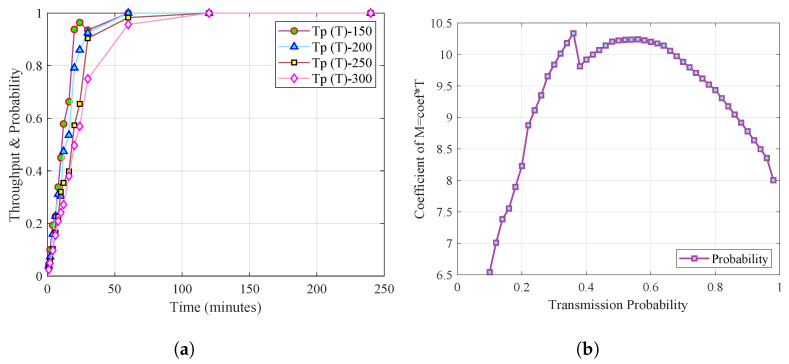
Cognitive channel transmission opportunity. (**a**) Throughput and probability vs. time; (**b**) transmission probability.

Figure 4a shows the timeline’s throughput relationship and transmission probability. It is evident that as time progresses, there is a relationship between the throughput and transmission probability equalizing from period 50 onwards. This effect is due to network saturation and the scarcity of available channels.

Figure 4b shows the relationship between the smart meters, the throughput, and the transmission probability. As a coefficient, it can be seen how the probability decreases notably when the intelligent meters and time increase. However, since the probability of transmission is probabilistic, there may be peaks in uncertain scenarios in which the likelihood of successful transmission may increase or decrease unpredictably. These uncertain scenarios can occur due to various factors, such as changes in network traffic or environmental conditions, which can affect the probability of transmission. Therefore, it is essential to consider the probabilistic nature of the transmission probability when analyzing network performance under different conditions.

**Figure 5:**
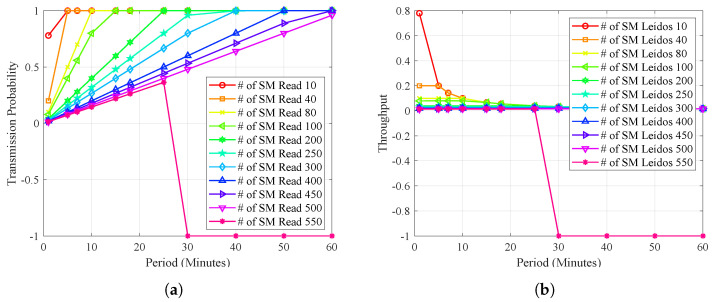
Behavior over time—channel assignment. (**a**) Transmission probability/period; (**b**) throughput/time.

Network saturation, congestion, or channel scarcity may lead to data loss, which could compromise the system’s overall performance. In Figure 5a, the behavior of the transmission probability of smart meters is displayed as a function of time. The results show that the transmission probability decreases as the smart meters increase, particularly within a shorter timeframe. Furthermore, a higher number of smart meters necessitates a more extended period to attain the desired transmission probability, which is critical to ensure that data loss does not occur.

As depicted in Figure 5b, the throughput is highest when the number of smart meters is small. Conversely, the throughput decreases as the number of smart meters increases. This trend continues as the timeline progresses, indicating that even with an increase in time, the throughput does not improve. Ultimately, the network becomes saturated, with no available channels, leading to a decline in throughput.

**Figure 6:**
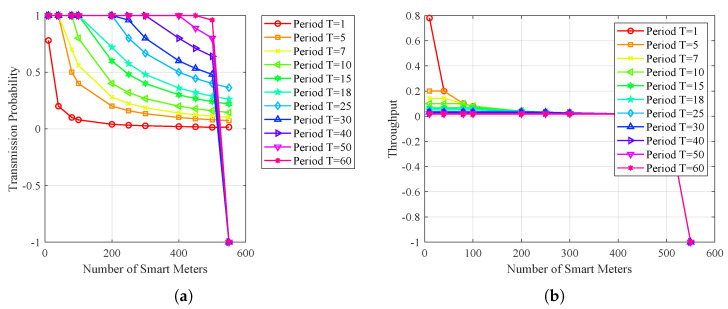
Opportunistic transmission to serve smart meters. (**a**) Transmission probability/# smart meters; (**b**) smart meter reading period (T).

Figure 6a illustrates the relationship between the transmission probability and the number of smart meters. The graph indicates that the transmission probability within a short period approaches zero with an increase in smart meters. Conversely, the likelihood of transmission is higher when the number of smart meters is small in a short period.

Figure 6b shows the throughput of smart meters. The results indicate that the throughput is higher when the number of smart meters is small within a short period. However, network saturation is reached as the number of smart meters increases, and the throughput does not improve.

**Figure 7:**
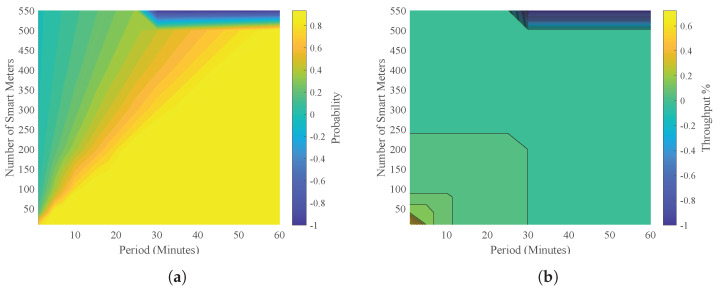
Impact of the number of smart meters to receive a free channel. (**a**) Relationship between # of smart meters and their probability of reading over time; (**b**) relationship between # of smart meters and throughput over time.

Figure 7a shows the analysis over time, where it can be seen that when the number of smart meters is not considerable and there is a short period, there is a low probability of a smart meter being read. However, the likelihood of reading increases when the timeline advances and the number of smart meters increases.

Figure 7b shows the behavior over time as the number of smart meters expected to be served increases and the throughput decreases. Thus, it is evident that the throughput is high when the number of smart meters is small.

## 5. Conclusions and Discussion

The presented algorithm, which focuses on finding channel matches for traffic generated by smart meters, enables the identification of usage trends and periods during which data transmission from smart meters, such as those for drinking water, electricity, or gas services, to the nearest cellular radio base can be ensured. The study highlights the potential coexistence of heterogeneous users from the primary operator and new secondary users, such as smart meters. Additionally, a mobile virtual network operator (MVNO) dedicated to innovative metering tasks could provide feasibility for primary spectrum use, eliminating the need for new infrastructure and reducing investment, maintenance, and scalability costs for metering services.

As a result, a C-MVNO with its cognitive stage could minimize costs by utilizing the spectrum opportunistically. However, if another cellular service company also uses an MVNO, detecting available white holes for a competent metering service becomes more complex due to increased channel competition. Therefore, leasing MVNO channels is essential to guarantee the availability of white holes for smart metering amidst the uncertainty of finding them.

This study has presented a simulation model for channel allocation in smart meter networks. Four main parameters have been considered: the maximum number of smart meters in a cell, the number of fixed and random channels reserved for the main grid operator and the virtual operator, and the meter reading period. It has been shown that there is a relationship between these parameters and the probability of transmission of smart meters in a short period.

Simulation has shown that more time is required to achieve an adequate transmission probability when the number of smart meters is high. In addition, when the number of random channels reserved for the virtual operator is low, congestion problems may be experienced in the network.

This simulation model may be helpful for network operators who wish to improve channel allocation in smart metering networks and ensure adequate transmission of metering data. In conclusion, this study has provided valuable information on smart metering channel allocation and has demonstrated that a simulation model is a valuable tool for analysis and improvement.

Dedicating smart metering tasks of different services to the cellular network undoubtedly reduces costs due to not deploying a dedicated wireless network. It is essential to remember that penetration may not reach 100% across the country, but it has more satisfactory coverage than a new network. With the advances in artificial intelligence, future work is foreseen wherein the network learns the times at which channels are available and thus achieves a lower cost but reliable, intelligent measurement. 

## Figures and Tables

**Table 1:** Summary of works related to peer instruction and learning engineering.

Work	Problem			Constraint				Proposal	
Author	Resource Allocation	Scheduling	Matching	Channels	Random Channel	Cost	Optimization/Heuristic	Channel Allocation	Cost
Dab, 2022 [12]	✠	✠		✠		✠	✠	✠	✠
Heng, 2022 [4]			✠			✠			✠
Wang, 2022 [2]	✠		✠	✠			✠	✠	
Malik, 2022 [7]	✠		✠	✠			✠		
Panda, 2022 [5]	✠	✠		✠			✠	✠	
Abbass, 2022 [3]	✠		✠		✠	✠	✠	✠	✠
Ivanov, 2022 [13]	✠	✠	✠			✠			✠
Taskou, 2022 [6]	✠		✠		✠	✠	✠		✠
Amer, 2021 [15]	✠		✠	✠	✠	✠	✠	✠	✠
Moon, 2021 [16]			✠	✠	✠	✠	✠		✠
**Present Work**	✠	✠	✠	✠	✠	✠	✠	✠	✠

**Table 2:** Notations used in this article.

Nomenclature	Description
*M*	Represents the number of smart meters served in the system
*N*	Represents the number of wireless channels available in the system
*T*	SM reading time intervals
*W*	Window length
*Z*	Quantity set of transmission frames
*F*	Transmission frames
TH	Throughput
$N_{r}$	Reserved channels
$N_{f}$	Fixed channels
$N_{ra}$	Random channels
$n_{c}$	Used channels
$n_{a}^{*}$	Smart meters that transmit
$n_{a}$	Active smart meters
$t_{k}$	Used channels
$x_{h,t,k}$	Scheduling in each frame
*h*	Channel
*t*	Time slot
$t_{f}$	Time slot related to frame
*k*	*k*th smart meter
$w_{k}$	Fair weight
$w_{k}^{t}$	Weight depending on distance of time
$w_{k}^{h}$	Weight depending on chosen channel

## Data Availability

Not applicable.
